# Functional Specialization Among Members Of Knickkopf Family Of Proteins In Insect Cuticle Organization

**DOI:** 10.1371/journal.pgen.1004537

**Published:** 2014-08-21

**Authors:** Sujata S. Chaudhari, Bernard Moussian, Charles A. Specht, Yasuyuki Arakane, Karl J. Kramer, Richard W. Beeman, Subbaratnam Muthukrishnan

**Affiliations:** 1 Department of Biochemistry and Molecular Biophysics, Kansas State University, Manhattan, Kansas, United States of America; 2 Department of Animal Genetics, Interfaculty Institute for Cell Biology, University of Tübingen, Tübingen, Germany; 3 Department of Medicine, University of Massachusetts, Worcester, Massachusetts, United States of America; 4 Division of Plant Biotechnology, Chonnam National University, Gwangju, Korea; 5 Department of Entomology, Kansas State University, Manhattan, Kansas, United States of America; The University of North Carolina at Chapel Hill, United States of America

## Abstract

Our recent study on the functional analysis of the Knickkopf protein from *T. castaneum* (TcKnk), indicated a novel role for this protein in protection of chitin from degradation by chitinases. Knk is also required for the laminar organization of chitin in the procuticle. During a bioinformatics search using this protein sequence as the query, we discovered the existence of a small family of three *Knk*-like genes (including the prototypical *TcKnk*) in the *T. castaneum* genome as well as in all insects with completed genome assemblies. The two additional *Knk*-like genes have been named *TcKnk2* and *TcKnk3*. Further complexity arises as a result of alternative splicing and alternative polyadenylation of transcripts of *TcKnk3*, leading to the production of three transcripts (and by inference, three proteins) from this gene. These transcripts are named *TcKnk3-Full Length* (*TcKnk3-FL*), *TcKnk3-5′* and *TcKnk3-3′*. All three *Knk*-family genes appear to have essential and non-redundant functions. RNAi for *TcKnk* led to developmental arrest at every molt, while down-regulation of either *TcKnk2* or one of the three *TcKnk3* transcripts (*TcKnk3*-3′) resulted in specific molting arrest only at the pharate adult stage. All three *Knk* genes appear to influence the total chitin content at the pharate adult stage, but to variable extents. While *TcKnk* contributes mostly to the stability and laminar organization of chitin in the elytral and body wall procuticles, proteins encoded by *TcKnk2* and *TcKnk3*-*3′* transcripts appear to be required for the integrity of the body wall denticles and tracheal taenidia, but not the elytral and body wall procuticles. Thus, the three members of the Knk-family of proteins perform different essential functions in cuticle formation at different developmental stages and in different parts of the insect anatomy.

## Author Summary

We have identified two additional members of the family of Knickkopf (Knk)-like proteins in the genome of the red flour beetle as well as in all insect species with completely sequenced genomes. The previously characterized member of this family, TcKnk, protects chitin in the newly forming cuticle (exoskeleton) from degradation by the chitinase enzyme present in the molting fluid. Knk is also required for the laminar organization of the chitin polymer in the cuticle. The two newly identified members of this family, TcKnk2 and TcKnk3, have distinctly different but related functions. They are essential for adult morphogenesis including specialized “Velcro-like” cuticular denticles found in the lateral body wall, as well as the proper development of the tracheal lining. The *TcKnk3* gene gives rise to multiple transcripts as a result of alternative polyadenylation and/or splicing, but only one of these transcripts is essential for adult development.

## Introduction

Chitin, a homopolymer of β-1,4 linked N-acetyl glucosamine units, is an essential component of the extracellular matrix of insect cuticle [1]. Chitin, the major component of the procuticle, is synthesized by the integral membrane protein, chitin synthase-A (Chs-A) and is deposited outside of the cell in the form of bundles of fibers [2],[3]. Several such bundles of nanofibers and proteins are then arranged in the form of a chitin/protein sheet, or lamina [4],[5]. In some parts of the procuticle, successive layers of chitin sheets are deposited in such a way that the horizontal axes of adjacent laminae follow a helical path [4]. Several groups of cuticle-associated proteins have been implicated in organizing the cuticle into a complex multi-layered structure with distinctly different properties in different parts of the insect's body plan [6],[7]. The role, if any, of specific cuticular and epidermal cell plasma membrane proteins in organizing chitin laminae into such helicoidal or orthogonal bundles has not been investigated in detail.

During development, in addition to cuticle growth, insects have to undergo a process of molting, during which old cuticle is replaced by a new one. Chitinases present in the molting fluid degrade chitin from the old cuticle, providing substrate for the new cuticle synthesis [8],[9],[10]. Our recent study with the red flour beetle, *Tribolium castaneum*, has shown co-localization of chitinase (chitinase-5) with chitin in the newly synthesized procuticle leading to the paradoxical situation where the nascent chitin needs protection from degradation by chitinases present in the molting fluid [11]. The presence of a chitin-binding protein, Knickkopf (Knk), in the developing new procuticle was shown to be important for protecting chitin from degradation by active chitinases [11]. *Knk* was initially identified in a search for mutants defective in cuticle integrity in developing embryos of *Drosophila melanogaster* [12] and molecularly characterized several years later [13]. Although the exact mechanism of protection of chitin by Knk remains unclear, its ability to bind to chitin was predicted to lead to a masking effect that prevents chitin from degradation by chitinases. Furthermore, Knk was also shown to be important for cuticle integrity and laminar organization of the embryonic procuticular chitin in *D. melanogaster* [14] and *T. castaneum* [11],[15]. The trafficking of Knk to the procuticle requires the participation of Retroactive, a protein belonging to the Ly-6 family, whose members are involved in a variey of protein-protein interactions [15].

In the current study, we describe the identification of two paralogous *Knk*-like genes, *TcKnk2* and *TcKnk3*, in the genome of *T. castaneum* and determine their patterns of expression during development and in different tissues including midgut, hindgut and carcass. RNA interference (RNAi) studies reveal important roles of these two additional *Knk*-family genes in embryonic as well as post-embryonic stages of development that are distinctly different from those of Knk. The protein products of these two genes are required for the maintenance of the integrity of cuticular structures in the body wall denticles and tracheal taenidia. A bioinformatics search of genomes of other insects indicate the presence of orthologs of *TcKnk2* and *TcKnk3* in all other insect species examined, suggesting conserved essential functions for these orthologs during insect cuticle morphogenesis in specialized cuticular structures.

## Results

### Identification Of *Tcknk*-Like Genes From *T. Castaneum* Genome

**Figure 1:**
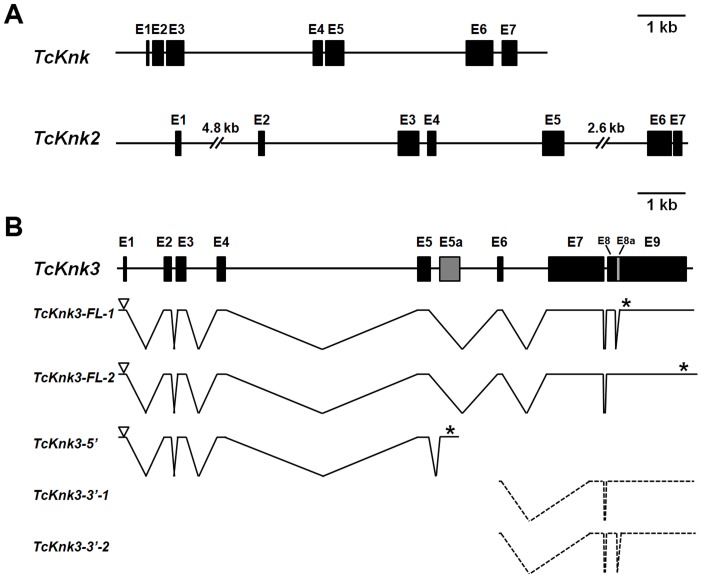
Schematic diagram of the exon–intron organizations of the putative *Tribolium castaneum Knk*-family genes. (A–B) The exon–intron organization of each *TcKnk*-family- gene was determined by sequence comparison between genomic sequence and the longest available cDNA sequence. (A) Gene organization of *TcKnk* and *TcKnk2*; (B) Alternatively spliced forms of *TcKnk3* (*TcKnk3-FL-1*, *TcKnk3-FL-2*, *TcKnk3-5′*, *TcKnk3-3′-1* and *TcKnk3-3′-2*). Black closed boxes indicate exons (E) and lines indicate the introns. The gray colored closed box indicates exon 5a for *TcKnk3-5′*. The open arrowheads represent the open reading frame start codons and * indicates the stop codons. The TcKnk3-3′-transcripts are indicated using broken lines because the precise location of the 5′-ends are unknown.

Two previously uncharacterized homologs of the *TcKnk* (LOC655087) (JN314843) gene were detected in a search of the *T. castaneum* genome using the NCBI TBLASTN program and TcKnk protein sequence as the query. These *TcKnk*-like genes, which we designate as *TcKnk2* (LOC 661990) (KF475699) and *TcKnk3* (LOC 657143) (KF475700), map on linkage group 9, at positions 11.0 cM and 34.1 cM, respectively. *TcKnk* maps to position 34.1 (same recombinational map position as *TcKnk3*). These two genes are closely linked on chromosome 9 and are separated by only 330 kbp of intervening sequences. Comparison of the sequence of a putative full length *TcKnk2* cDNA clone with the genomic sequence for *TcKnk2* indicated that it is composed of seven exons capable of encoding a protein of 70.56 kDa with 632 amino acids (Fig. 1A).

### *Tcknk3* Gene Contains An Additional Exon

A similar strategy utilized to clone cDNAs for *TcKnk3* indicated the presence of multiple transcripts for this gene. By RT-PCR using a pair of primers located at the 5′- and 3′-ends of the deduced *TcKnk3* mRNA and cDNA from the pharate adult stage as template, we obtained a long cDNA fragment with a size of ∼4 kb. We cloned this cDNA fragment and randomly chose two clones for sequencing. Sequence comparisons of cDNA clones (both had exactly the same sequence) and the mRNA sequence predicted by the NCBI gene model for this gene (*XM_963619* with 8 exons) indicated that they were in agreement except for the absence of a 55 nucleotide-long stretch in the last exon in these two cDNA clones. We have designated this “missing” stretch as “exon 8a” and the rest of the last exon as exon 9. We have named the transcript lacking this exon as *TcKnk3-Full Length-1* (*TcKnk3-FL-1*) (KF475700) (Fig. 1B). Inspection of the sequences flanking this 55 nucleotides-long presumptive exon 8a indicated that it has the potential to be an intron because it begins with 5′-GT— and ends in —CAG-3′ as expected of typical introns. To determine whether some *TcKnk3* transcripts lack this exon 8a sequence as predicted by the NCBI model, we used the same pharate adult cDNA template that was used to obtain the two long cDNA clones for additional PCR reactions using primers flanking exon 8a. We could obtain two amplified DNA fragments differing in size using these primers (Fig. S1). Sequencing of the larger fragment indicated that it had all 55 nucleotides of “exon 8a” sequences. We have named this transcript as *TcKnk3-Full Length-2* (*TcKnk3-FL-2*) (KF475701) (Fig. 1B). The relative abundances of these two PCR amplification products indicated that the transcripts without exon 8a predominate at the pharate adult stage (Fig. S1 A). Therefore, this 55 nucleotide stretch is indeed present only in a minority of mature transcripts, at least at this stage of development. Inclusion of this intron results in the read-through of exon 8a, a shift in reading frame, and extension of the ORF to a stop codon farther down-stream, leading to a much larger protein. The predicted lengths of TcKnk3-FL-1 and TcKnk3-FL-2 are 731 and 1205 amino acids, respectively. They share the first 728 amino acids starting from the N-terminus, differing only in the C-terminal region. Fig. S2 shows an alignment of the amino acid sequences of the Knk3 proteins encoded by these two transcripts.

### Alternative Splicing/polyadenylation Yields A Shorter Transcript Derived From The 5′-Part Of *Tcknk3* Gene

3′-RACE using a forward primer in exon 5 and oligo (dT) as the reverse primer revealed the presence of polyadenylated RNAs with two distinct sizes. One had a size of ∼4 kb consistent with that predicted from the gene model proposed in Fig. 1B, while the other was much shorter than the full length mRNAs for *TcKnk3* (with or without exon 8a). We have cloned the cDNA corresponding to this short transcript as described in the Materials and Methods section and named this transcript *TcKnk3-5′* (KF475702). *TcKnk3-5′* cDNA is 1942 nucleotides long and includes an ORF of 1209 nucleotides, which encodes a protein of 403 amino acids (45.6 kDa) and a pI of 9.28. The N-terminal 263 amino acid sequence of this encoded protein was identical to that of the full-length protein predicted by the NCBI gene model, but included an additional 141 amino acids at its C-terminus not present in the predicted products of the longer clones. Nucleotide sequence comparisons with the *TcKnk3* genomic sequence indicated that *TcKnk3-5′* cDNA is the result of an alternative splicing event that led to the inclusion of an exon (named exon 5a) and a polyadenylation site encoded within intron 5 of the NCBI gene model. Indeed, there is a polyadenylation signal 26 nucleotides upstream of the poly-A tail of this shorter 5′-transcript. The inclusion of exon 5a, which is 1156 nucleotides long, resulted in the translation of the 141 codons not present in the two full-length mRNAs (FL-1 and FL-2). This RNA contains in addition to the first 5 exons of *TcKnk*, an additional exon that we have designated as “exon 5a” with a 3′-UTR of 733 nucleotides (including the stop codon but excluding the poly A tail) (Fig. 1B).

### Northern Blot Analysis Reveals The Presence Of An Additional Transcript From The 3′-Half Of The *Tcknk3* Gene

**Figure 2:**
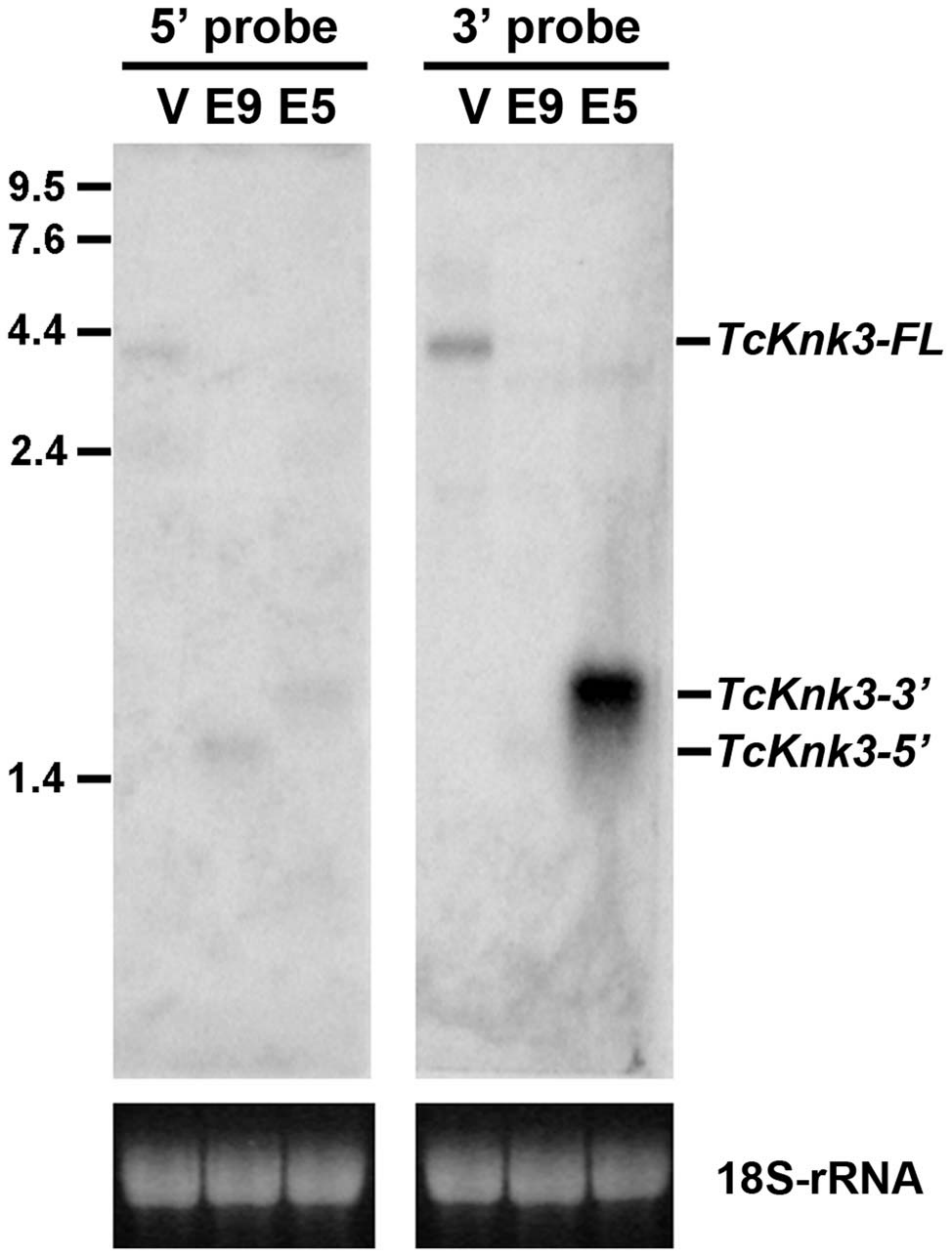
Northern blot analysis of *TcKnk3* transcripts using ^32^P-labeled 5′-terminal and 3′-terminal probes. Ten µg of extracted RNA from: V, *TcVer*; E9, *TcKnk3*-exon 9; E5, *TcKnk3*-exon 5 specific dsRNA-treated pharate adult insects was loaded onto an 1.5% agarose gel, transferred on to the nitrocellulose membrane and hybridized with ^32−^P-labeled 5′-terminal or 3′-terminal probe as described in Materials and Methods. There is a small amount of cross-hybridization of the 5′-probe and the RNA that gives a strong signal with the 3′-probe (presumed 3′-transcript; left panel, lane marked E5). The indicated sizes of the bands were based on the length of the cloned fragments and assuming a poly-A tail of ∼200 nucleotides.

To confirm the presence of the long and short transcripts identified above as well as to investigate the possibility of additional transcripts for the *TcKnk3* gene due to alternative splicing and/or polyadenylation, we performed a Northern blot analysis using ^32^P-labeled probes derived from the 5′- and 3′- regions of the *TcKnk3* gene as described in the Materials and Methods section. We anticipated that our chance to detect minor transcripts corresponding to the 5′- or 3′-regions of the gene could be enhanced by selectively down-regulating the steady state levels of one or more of the above-described transcripts using dsRNAs targeting exons either in the 5′- half or the 3′-half of this gene. Aliquots (10 µg) of total RNA extracted from pharate adult insects injected with control (*TcVer*), *TcKnk3* exon 9 or *TcKnk3* exon 5 dsRNA were used for Northern blot analysis (Fig. 2). Hybridization with the ^32^P-labeled 5′-probe (exon1-exon5 region) detected a ∼4 kb transcript in control RNA (extracted from *TcVer* dsRNA-treated insects), a result consistent with the sizes of the two *TcKnk3-FL* cDNA clones that we have sequenced (Fig. 1B). No other transcripts with smaller sizes were detected. However, in RNA extracted at the same developmental stage from animals treated with exon 9 dsRNA (designed to suppress the full length transcript), the 5′-probe detected only the shorter *TcKnk3-5′* transcript. The absence of the full-length (*TcKnk3-FL*) transcript in this RNA preparation confirmed that we are indeed detecting only the *TcKnk3*-specific transcripts and not transcript of closely related *TcKnk* and *TcKnk2* genes. In RNA from animals treated with dsRNA for exon 5, neither the full-length 4 kb transcript nor the *TcKnk3-*5′ transcript were detected as expected, but surprisingly a slightly larger band (>2 kb) was detected. In a second experiment with a duplicate blot of the same three RNAs, a ^32^P-labeled *TcKnk3*-3′ fragment was used as the hybridization probe. The 3′-probe also detected the *TcKnk3-FL* transcript in the control RNA and its level was undetected after administration of exon 9-specific dsRNA as expected. Once again, the shorter transcripts were not detected in this RNA. Surprisingly, there was a strong autoradiographic band corresponding to the >2 kb-long transcript in the blot probed with the 3′-probe. Compared to the control RNA in which the 3′-transcripts were undetectable (lane labeled V), there was substantial up-regulation of the steady-state level of this transcript when the transcripts for the full-length and *5′-TcKnk3* transcripts were down-regulated by exon 5-specific dsRNA (lane labeled E5, Fig. 2). We presume that this transcript is derived predominantly from the 3′-half of the *TcKnk3* gene, as it does not hybridize with the 5′-probe. The largest ∼4 kb band (in lane labeled V) is presumably a mixture of molecules with or without exon 8a sequences. There were some minor bands in some samples. We presume that these represent pre-mRNA or additional alternative splicing products. Taken together, these results provide further evidence that the *TcKnk3* gene has the potential to yield at least five different forms of transcripts that differ in sizes and exon composition and that their relative abundances can be altered under appropriate conditions.

### *Knk*-Family Genes Are Present In Several Orders Of Insects

**Figure 3:**
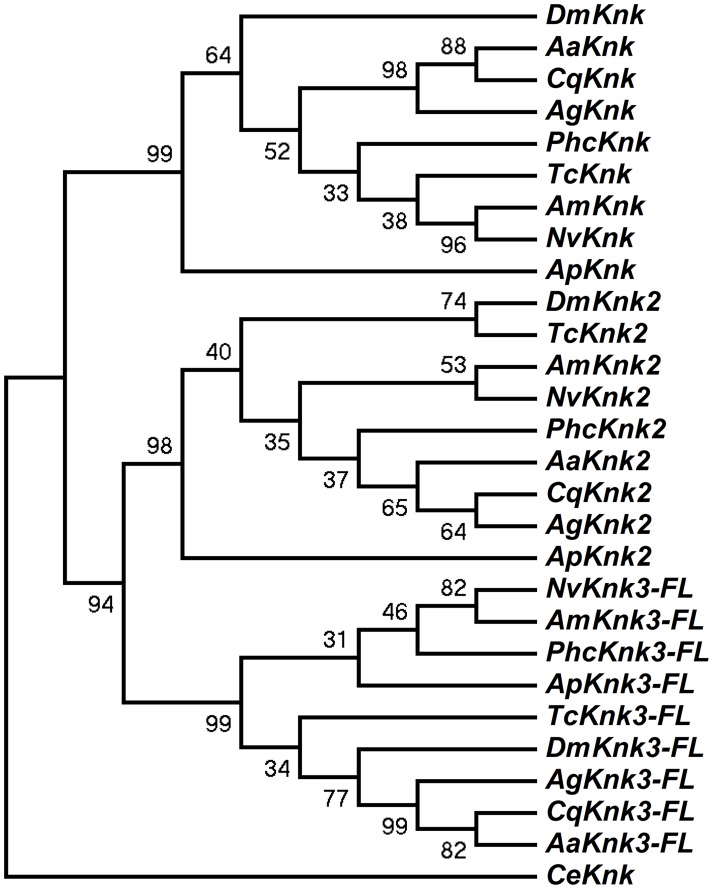
Phylogenetic analysis of insect TcKnk2 and TcKnk3. Protein sequence predicted from the TcKnk3- full length transcript without exon 8a was used for analysis. MEGA 4.0 was used to construct the phylogenetic tree using the neighbor joining method [16]. Bootstrap analyses from 5000 replications are shown by each branch. *Drosophila melanogaster* (fruit fly) (Knk2, NP_001097889.2; Knk3, NP_001027171.1); *Anopheles gambiae* (African malaria mosquito) (Knk2, XP_308115.4; Knk3, XP_313250.2); *Aedes aegypti* (yellow fever mosquito) (Knk2, XP_001657415.1; Knk3, XP_001657668.1); *Culex quinquefasciatus* (southern house mosquito) (Knk2, XP_001845444.1; Knk3, XP_001864909.1); *Pediculus humanus corporis* (head louse) (Knk2, XP_002429468.1; Knk3, XP_002425509.1); *Tribolium castaneum* (red flour beetle) (Knk2, XP_973211.1; Knk3, XP_968712.2); *Apis mellifera* (honey bee) (Knk2, XP_393508.4; Knk3, XP_003250319.1); *Nasonia vitripennis* (parasitic wasp) (Knk2, XP_001602771.1; Knk3, XP_001606495.2) and *Acyrthosiphon pisum* (pea aphid) (Knk2, XP_001946688.2; Knk3, XP_003241759.1); *Caenorhabditis elegans* (roundworm) (NP_508959.1).

The identification of two additional paralogous genes encoding Knk-family proteins in the *T. castaneum* genome prompted us to investigate whether *Knk*-family genes are present in other insect orders as well. A search of sequence databases of several insects with fully sequenced genomes including those of *D. melanogaster*, *A. gambiae*, *A. aegypti*, *C. quinquefasciatus*, *A. mellifera*, *A. pisum*, *N. vitripennis* and *P. humanus corporis* indicated that orthologs of *TcKnk2* and *TcKnk3* are present in all these genomes (Fig. 3). The predicted sequences of the full-length Knk-family proteins from several insects were used to construct a phylogenetic tree using the neighbor-joining method [16]. This analysis indicates that the Knk-family proteins from all of these insect species neatly separate into three clades, with each clade having one *Knk* gene from each insect. Conservation of all three TcKnk-family proteins in different insect species indicates an essential role for these proteins presumably for the development of the chitinous exoskeleton in many insect species. However, our search failed to identify orthologs in non-insect arthropods such as the water flea and the deer tick, nor were any found in the genomes of the sea urchin and nematode, even though all of them do have orthologs of *TcKnk* [11].

### Domain Organization And Phylogenetic Analysis Of Knk Family Of Proteins

**Figure 4:**
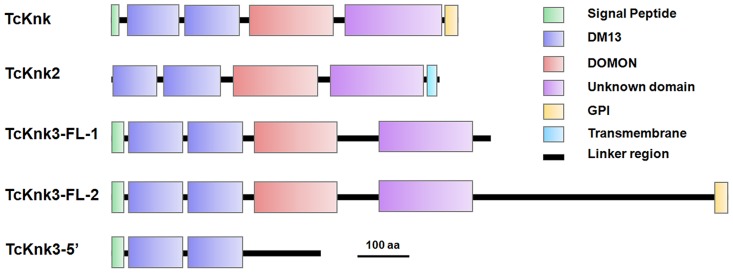
Domain architecture of putative TcKnk-family proteins from *Tribolium castaneum*. Domain analysis was done using SMART protein. Red: Signal peptide; Blue: DM13 domain; Purple: DM13 domain; Green: DOMON domain; Yellow: GPI anchor; Cyan Blue: Trans-membrane domain.

Both *DmKnk* and its ortholog, *TcKnk*, are predicted to encode putative C-terminal GPI-anchored proteins containing two tandem N-terminal DM13 domains and a central dopamine β-monooxygenase N-terminal-like (DOMON) domain followed by a C-terminal sequence that remains uncharacterized [14]. The domain organizations of TcKnk-family of proteins were predicted using the SMART protein database. Domain analysis indicated that all three members of the *TcKnk*-family of genes are capable of encoding proteins with two DM13 domains and a DOMON domain (Fig. 4). These domains are followed by a middle region (∼100 amino acids) of low sequence similarity followed by a C-terminal stretch of about 200 amino acids, which is highly conserved among these three Knk-family proteins (Fig. S3). This C-terminal stretch appears to be unrelated to any of the well characterized protein domains currently in the SMART database.

The three proteins of the Knk family differ with respect to the presence or absence of membrane-anchoring sequences at their carboxyl termini. While TcKnk and TcKnk3-FL-2 proteins are predicted to have a GPI anchor sequence at their C-termini, TcKnk2 is predicted to have a trans-membrane segment at the C-terminal end (Fig. 4). The protein encoded by the shorter *TcKnk*3-5′-transcript has the two DM13 domains but is missing the DOMON domain and all of the downstream sequences. It has no predicted GPI anchor or TM segments (Fig. 4). Figure S3 shows an alignment of these three TcKnk-family full-length proteins, which emphasizes the similarities in the N-terminal and C-terminal regions and differences in the middle parts of these proteins.

### Developmental Stage And Tissue-Specific Expression Profiles Of *Tcknk2* And *Tcknk3*

**Figure 5:**
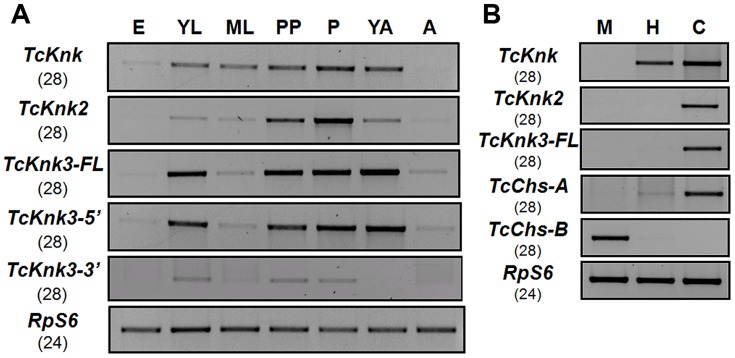
Developmental stage-specific and tissue-specific expression of *TcKnk*-family genes by RT-PCR. (A) Developmental expression profiles of *TcKnk* (includes data on *TcKnk1* taken from [11]) for the purpose of comparison, *TcKnk2*, *TcKnk3-FL*, *TcKnk3-5′* and *TcKnk3-3′* transcripts. cDNAs were prepared from total RNA extracted from whole insects at several developmental stages including E, embryos; YL, young larvae (penultimate instar or younger); ML, mature larvae; PP, pharate pupae; P, pupae; YA, young adults (0 d- old); A, mature adults (10 d-old). (B) Tissue-specific expression of *TcKnk*-family genes in the feeding stage last instar larvae. M, midgut; H, hindgut; and C, carcass (whole body without gut). *T. castaneum* ribosomal protein-S6 (*TcRpS6*) was used as internal loading control for RT-PCR. Results are from 28 and 24 cycles of RT-PCR for *TcKnk*-family *genes* and *TcRpS6*, respectively. *TcChs-A* (chitin synthase-A) (epidermis-specific) and *TcChs-B* (chitin synthase-B) (midgut-specific) expression was also measured to rule out cross contamination with RNA from non-targeted tissues.

To determine whether there are differences in the expression patterns of *TcKnk*-family genes during *T. castaneum* development, we analyzed the steady-state levels of *TcKnk2*, *TcKnk3-FL-1*, *TcKnk3-FL-2*, *TcKnk3-5′* and *TcKnk3-3′* transcripts using cDNA templates prepared from RNA extracted from embryos, young larvae, mature larvae, pharate pupae, pupae, young adults (0 d-old) and mature adults (10 d-old). *TcKnk2* transcripts were detected at all stages of insect development except the embryonic stage, with the highest expression levels being found in the pupal stage (Fig. 5A). *TcKnk3-FL* as well as *TcKnk3-5′* transcripts were also barely detectable in embryos but were abundant in young larvae, pharate pupae, pupae and young adults (Fig. 5A). These results are almost identical to those for *TcKnk* expression, except that *TcKnk3* transcripts peaked in young adults rather than in pupae. Transcripts for both of these genes (as well as *TcKnk*) were detected in carcass and hindgut, but not in midgut tissue, consistent with a role for these proteins in cuticle-forming tissues but not in peritrophic matrix (PM)-forming tissues (Fig. 5B).

### Does *Tcknk2* Contribute To Molting Of *T. Castaneum*?

**Figure 6:**
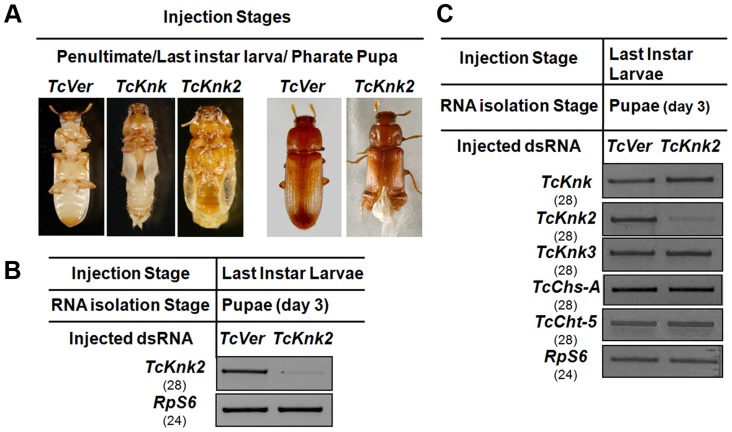
Effect of *TcKnk2* dsRNA-treatment on the development of *T. castaneum*. (A) Injection of dsRNA for *TcKnk2* into penultimate instar larvae, last instar larvae and pharate pupae (n = 60) led to lethal phenotype at pupal-adult molt (∼55%) and ∼15% adult hypomorphic phenotype with split elytra. (B) Specific down-regulation of *TcKnk2* transcripts by RNAi. dsRNAs (200 ng per insect) for *TcKnk2* were injected into pharate pupae. Three days after injection, total RNA was extracted from whole insects of at pupal stage day 3 (n = 3) and used for cDNA synthesis. (C) Specificity of *TcKnk2* dsRNA-treatment. Effect of *TcKnk2* dsRNA-treatment on transcripts for *TcKnk*, *TcKnk3* and other genes involved in chitin metabolism such as *TcChs-A* and *TcCht5* was checked by RT-PCR. *T. castaneum ribosomal protein-S6* (*TcRPS6*) was used as internal loading control. dsRNA for *T. castaneum Vermilion* (*TcVer*) and *TcKnk* was injected as a control.

We have shown previously that RNAi of *TcKnk* results in arrest of insect development at every molt [11]. To determine whether the paralogous *TcKnk2* gene has any role in *T. castaneum* development and molting, we injected young larvae, last instar larvae and pharate pupae with two dsRNAs targeting two different regions of the *TcKnk2* gene (*dsTcKnk2*), but the results shown are from dsRNA1 (Table S1). *dsVermilion* (*dsTcVer*), a dsRNA targeted specifically against *tryptophan oxygenase*, a gene responsible for eye pigmentation in *T. castaneum*, was used as a control. About 55% of the insects subjected to *TcKnk2* dsRNA treatment at the young larval, last instar larval or pharate pupal stages exhibited lethal phenotypes at the pharate adult stage. There was no evidence of developmental arrest at the larval-larval or larval-pupal molts. At the pharate adult stage, the dsRNA-treated insects exhibited a clear molting defect as a result of an inability to shed the old pupal cuticle (Fig. 6A). The remaining insects metamorphosed into adults, but about 30% of the dsRNA-injected insects had a weaker phenotype. These insects exhibited a split wing phenotype as a result of an improper folding of the hindwings and elytra (Fig. 6A). All adults with this hypomorphic phenotype died within 10–15 days of adult emergence, whereas the remaining adults (∼15% of dsRNA-treated insects) were normal and showed no visible phenotype or mortality in comparison with control animals injected with dsRNA for *TcVer*. *TcKnk2* transcript levels were significantly down-regulated after dsRNA *TcKnk2* treatment in comparison with control dsRNA *TcVer*-injected insects (Fig. 6B).

The molting defect observed at the pharate adult stage after *TcKnk2* RNAi was similar to that of *TcKnk* or *TcChs-A* RNAi phenotypes. To determine whether this is due to cross-knockdown of transcripts of *TcKnk* or other genes involved in chitin metabolism such as *chitin synthase-A* (*TcChs-A*) or *chitinase-5* (*TcCht5*, which leads to developmental arrest at a slightly later pharate adult stage), we performed RT-PCR using cDNA prepared from RNA extracted from 3-d-old pupae (n = 4) after *TcKnk2* dsRNA treatment. RT-PCR using gene-specific primers confirmed specific knockdown of *TcKnk2* transcripts upon *TcKnk2* RNAi with no apparent decrease in the transcript levels for *TcKnk*, *TcKnk3*, *TcChs-A* or *TcCht5* (Fig. 6C). These results suggest that the observed phenotypes are due to depletion of *TcKnk2* transcripts and not the result of down regulation of transcripts for other genes of chitin metabolism studied here.

### Rnai Reveals That Only One Of The Alternatively Spliced Transcripts Of *Tcknk3* Is Essential For Adult Morphogenesis

**Figure 7:**
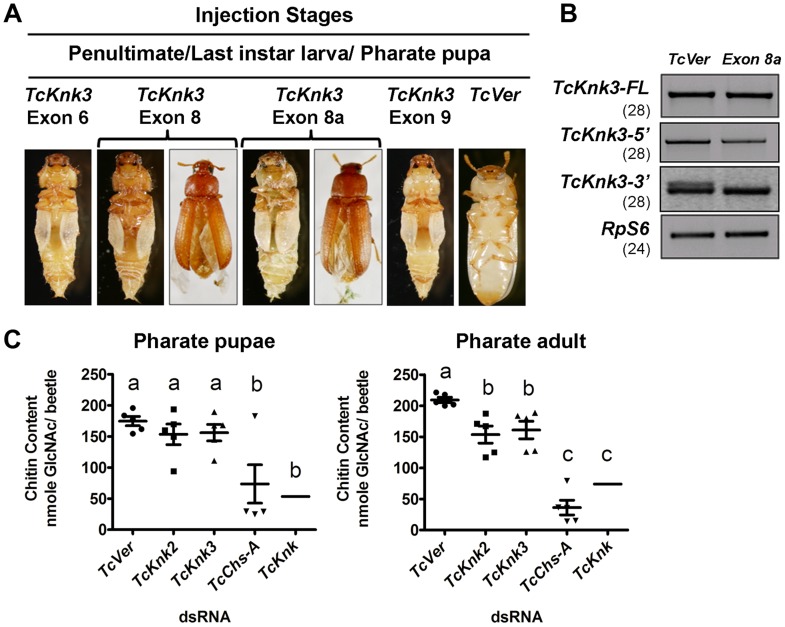
Effect of *TcKnk3* dsRNA-treatment on the development of *T. castaneum*. (A) dsRNAs specific for different exons and intron of *TcKnk3* (exon 6, exon 8, exon 9 and exon 8a) were injected into penultimate instar larvae, last instar larvae and pharate pupae. Approximately 60–100% of the dsRNA-treated insects showed lethal phenotype at pupal-adult molt. Exon 8 and exon 8a dsRNA-treatment led to a weaker phenotype with split elytra. (B) Specific down-regulation of *TcKnk3-3′* transcripts by RNAi. dsRNA (200 ng per insect n = 60) for *TcKnk3-3′* (corresponding to exon 8a) was injected into pharate pupae. Five days after injection, total RNA was extracted from pharate adult insects (n = 3) and used for cDNA synthesis. Depletion of different transcripts was checked by using pairs of transcript-specific primers (Table S1). For detection of transcripts with or without exon 8a, a pair of forward and reverse primers flanking exon 8a was used (see Table S1). *TcRPS6* was used as internal loading control. *TcVer* dsRNA was injected as a control. RT-PCR for the *TcRpS6* transcripts was carried our prior to these analyses to ensure that equal amounts of cDNA templates from different developmental stages were being used in these comparisons. Results are from 28, 28, 28 and 24 cycles of RT-PCR for *TcKnk3-FL TcKnk3*-*5′ and TcKnk3-3′* and *TcRpS6*, respectively. The RT-PCR products were run on separate gels for each RT-PCR product (with different sizes for each transcript), but the figure as shown is composite showing the relevant regions only to avoid “white space”. The grainy quality of the amplification products for *TcKnk3-3′* relative to the other RT-PCR products is due to different camera settings used to reveal the minor band from the alternatively spliced product with exon 8a sequences. (C) *TcKnk2* or *TcKnk3* (exon-9)-specific dsRNA was injected into last instar larvae and pharate pupae. Four to five days after injections, pharate pupal and pharate adult (n = 5) insects were collected for chitin content analysis by a modified Morgan–Elson method as described earlier [21]. dsRNA for *TcVer* and *TcChs-A* were injected as negative and positive controls, respectively. The mean chitin content for TcKnk dsRNA treated insects is adapted from previously published data [11].

The finding that there are multiple transcripts corresponding to the *TcKnk3* gene presented some challenges in determining their function by RNAi. dsRNAs corresponding to regions in several exons were designed to down-regulate selected or multiple transcript(s) as desired. A dsRNA corresponding to the 55 bp region of the “exon 8a” was designed to down-regulate only the transcripts with this sequence. These dsRNAs were injected into insects at young larval, last instar larval and pharate pupal stages of *T. castaneum* development. To determine the specificity and effectiveness of RNAi, we examined the levels of *TcKnk3-FL*, *TcKnk3-5′* and *TcKnk3-3′* transcripts after each dsRNA treatment using cDNAs prepared from RNA extracted from pharate adult insects (four days after dsRNA injections) utilizing appropriate forward and reverse primers. Significant depletion of the targeted transcript(s) was observed with each dsRNA treatment (Fig. S4). While the levels of *TcKnk3-FL* (with or without exon 8a) transcripts were significantly reduced after treatment with all of the dsRNAs tested, the *TcKnK3-5′* transcript was affected only by dsRNAs designed from exon 1 to exon 5 sequences but not by dsRNAs for downstream exons. dsRNA for exon 8a affected only those transcripts with exon 8a sequences, but not those without it, including the TcKnk-5′ transcript (Fig. 7B).

Despite substantial depletion of both *TcKnk3-FL* and *TcKnk3-5′* transcripts by dsRNA treatment targeting exon 5 (Fig. S4, Table S2), no visible phenotype or mortality was observed. All insects injected with this dsRNAs at any stage of development produced adults without molting defects or visible abnormalities. Unexpectedly, injection of exon 9-specific dsRNA that also led to a similar depletion of the *TcKnk3-FL* transcripts resulted in 100% mortality at the pharate adult stage of molting. No molting defects or abnormal phenotypes were observed during the earlier stages of development including larval-larval and larval-pupal molts. To understand the differences in the RNAi results from exon 5- versus exon 9-specific dsRNA treatments, we injected insects from different stages of development with dsRNAs specific for different exons including exon 1 (with the 5′-UTR region), exons 2 and 3 (spanning both exons), exon 6, exon 8 and exon 9. After injection of dsRNA for exon 1 or exons 2–3, insects developed normally into adults in comparison with control *dsRNA* (*TcVer*)-treated insects similar to those injected with dsRNA for exon 5. However, insects treated with either exon 6 or exon 8 dsRNA showed molting defects similar to those seen after exon 9-specific RNAi, resulting in mortalities of 100% and 82%, respectively, at the pharate adult stage of development (Fig. 7A). Even the survivors (18%) of the exon 8-specific dsRNA treatment exhibited a weak phenotype with split elytra as adults (Fig. 7A).

The failure of dsRNAs for the exons near the 5′-end of the *TcKnk3* gene (exon 1 through exon 5) to yield any visible alterations in phenotype in contrast with the effectiveness of the dsRNAs for the down-stream exons (exon 6 and downwards) indicated the possibility that the transcript derived from the 3′ region of the *TcKnk3* gene, which lacked sequences corresponding to several of the exons in the 5′-half of the gene may be the only one essential for insect survival. Presumably this is the same transcript that accumulates in the pharate adult stage when the 5′ and FL-transcripts are down-regulated by treatment with dsRNAs for the exon 5 (Fig. 2). Upon RNAi with dsRNAs for downstream exons, this short transcript might have been depleted leading to the observed phenotypes. Since we found exon 8a sequences in a minority of transcripts, we also investigated whether dsRNA for this exon could yield the same phenotype as exon 8-specific dsRNA. About 65% of the insects injected with exon 8a dsRNA exhibited mortality at the pharate adult stage and 15% of the surviving adults had a weaker phenotype similar to that observed after exons 6, 8 and 9 dsRNA treatments, indicating that transcripts with exon 8a are critically important for development and molting. RT-PCR analysis of total RNA from insects treated with dsRNA for exon 8a indicated that this treatment did not result in visible depletion of the full-length transcripts (Fig. 7B). RT-PCR analysis using primer pairs flanking exon 8a specifically designed to detect transcripts with and without exon 8a indicated that exon 8a dsRNA specifically depleted only the transcripts containing exon 8a without appearing to affect those without this sequence. These data further suggest that transcripts without exon 8a (whether full length or shorter transcripts) are not essential for survival of the insects during the pupal-adult transformation. From all of the data from multiple dsRNA treatments, we conclude that only the *TcKnk3*-*3′* shorter transcripts with the 55 nucleotides-long exon 8a sequences appear to be essential for survival and molting.

### The Splicing Of *Tcknk3*-Exon 8a Is Developmentally Regulated

RT-PCR reactions using RNA preparations made from insects at different developmental stages and a pair of forward and reverse primers flanking exon 8a revealed a variation in the relative abundance of transcripts, which differ in the presence or absence of exon 8a sequences. The smaller fragment (without exon 8a) was more abundant than the larger fragment in the RT-PCR products of RNA isolated at all pupal stages except on pupal day 2 when the relative abundance was reversed (Fig. S1B). The amount of the larger fragment (with the exon 8a sequence) was nearly constant during the pupal stage while the smaller transcript (without the exon 8a sequence) underwent dramatic changes in abundance. During the mid-pupal stage (P2), transcripts with exon 8a sequences predominate.

### Tcknk2 And Tcknk3 Are Required For Chitin Maintenance And Integrity Of Procuticular Chitin In *T. Castaneum*

Our recent work has uncovered an important role for TcKnk in protection of procuticular chitin from chitinases [11]. TcKnk has been shown to be important both for the maintenance of chitin levels and its laminar organization in the procuticle [11],[14]. To determine whether *TcKnk2* and *TcKnk3* genes have any roles in cuticular chitin maintenance, we performed total chitin content analysis of larvae treated with *TcKnk2*- and *TcKnk3-3′* (exon-9)-specific dsRNAs. Insects were collected at pharate pupal and pharate adult stages of development four to five days after dsRNA injections into either last instar larvae or pharate pupae. There was a significant decrease in chitin content after either *TcKnk2* or *TcKnk3-3′* dsRNA treatment at the pharate adult stage of development, but not at the pharate pupal stage, indicating an essential role for these two genes in cuticular chitin level maintenance specifically at the pharate adult stage of development (Fig. 7C). However, the decrease in chitin levels observed after RNAi for these two Knk paralogs was less substantial than that observed after RNAi for *TcKnk* [11].

**Figure 8:**
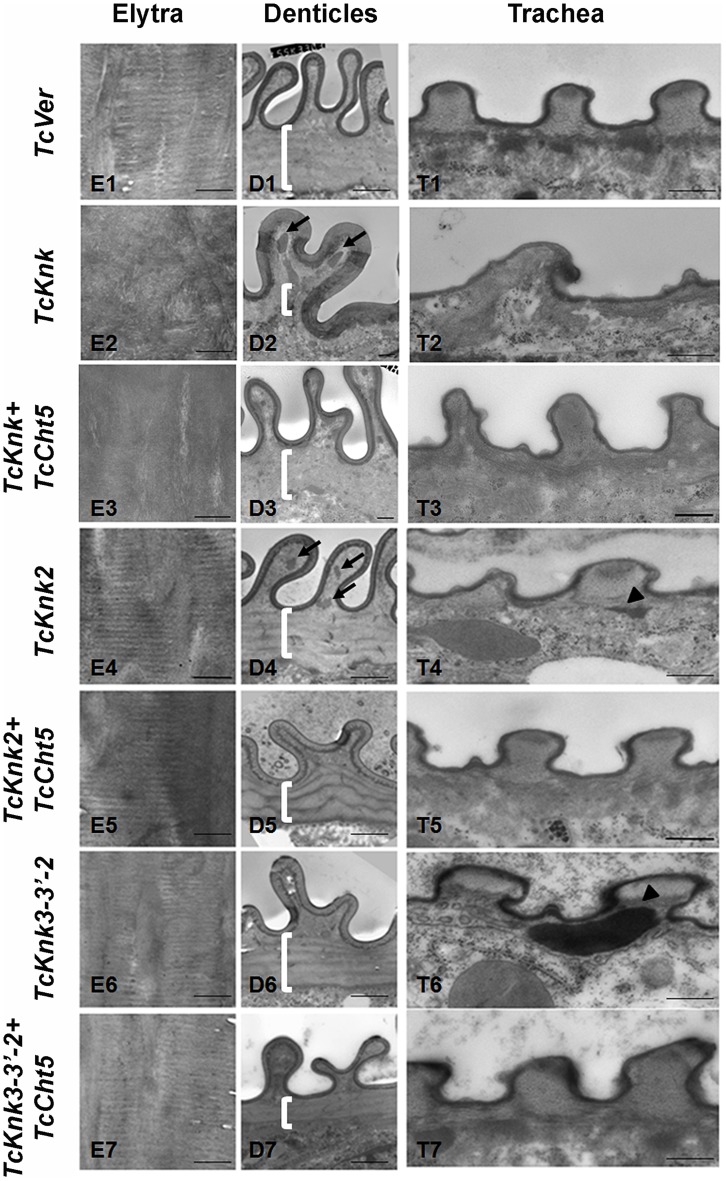
Transmission electron microscopic analysis of *TcKnk2* and *TcKnk3* (exon 9)-specific dsRNA-treated pharate adult elytra, lateral body wall denticles and tracheal taenidia. Larvae in the late stages of development were subjected to RNAi using the indicated dsRNA combinations as described in legend to Fig. 7. The dsRNA-treated insects were collected at the pharate adult stage and fixed for TEM analyses as described in the “Materials and methods” section. White brackets point to the area where laminae are found in *TcVer* control denticles (panel D1) but absent in corresponding sections from insects treated with dsRNA for *TcKnk* (panel D2) or *TcKnk* and *TcCht5* (panel D3). The arrows in panels D2 and D4 indicate electron-dense material accumulating under the bulges of Velcro-denticles; Arrowheads in the panels of tracheal sections (panels T4 and T6) indicate accumulation of electron-dense material under the taenidiae. They are absent in samples from insects treated with dsRNA both *TcKnk2* and *TcCht5* (panel T5) or with a mixture of dsRNAs for *TcKnk2* and *TcCht5* (panel T7). Scale bar, 500 nm.

To further determine the roles, if any, of *TcKnk*-family genes in organization of the procuticular chitin, we performed transmission electron microscopic (TEM) analysis of pharate adult elytral cuticle, lateral body wall denticle cuticle and the tracheae. TEM of elytra from control (*TcVer*)-dsRNA-treated insects revealed a horizontally arranged laminar organization of the procuticular chitin, that is, parallel to the apical surface of the epidermal cells (Fig. 8; panel E1). RNAi of *TcKnk* resulted in the loss of laminar organization of the elytral cuticle (Panel E2). A similar loss of laminar organization of chitin was also observed under the denticle-like structures associated with specific regions of the lateral body wall, which interlock with corresponding regions on the inner side of the elytra (compare panels D1 and D2), which we denote as “Velcro” (Arakane et al., unpublished data). These Velcro-like denticles have hooked structures and that are complementary to specialized structures found in specific regions of the elytra capable of interactions similar to fibers of “Velcro” that snap them together tightly. In addition, electron dense material accumulates under these folds (indicated by black arrows in Fig. 8). The tracheal taenidial cuticle was abnormal in shape (compare panel T1 and T2). Simultaneous down-regulation of chitinase 5 transcripts failed to ameliorate these morphological abnormalities brought about by RNAi for *Knk* (panels E3, D3 and T3). Thus RNAi of *TcKnk* affects not only the elytral and body wall cuticle as reported previously [11], but also other cuticles such as those associated with the tracheal cuticle and body wall “Velcro denticle” cuticle.

Similar TEM analyses were conducted after RNAi for *TcKnk2* and *TcKnk3* using abdominal sections of insects with the majority phenotype, i.e. insects that failed to expand their elytra and failed to contact their abdomen. However, no significant difference in the laminar architecture of elytral procuticular chitin in comparison with control insects was seen after *TcKnk2* and *TcKnk3-3′* dsRNA treatments (Fig. 8; compare panels E1, E4 and E6). Similarly, the laminar architecture of the body wall cuticle was also unchanged. In insects treated with *TcKnk2* dsRNA, a rather subtle phenotype is observed in the denticle-like structures associated with “Velcro denticles” (Fig. 8; panel D4). These “Velcro denticles” can be normally divided into two regions, the basal flat and the upper protruding region. The basal region procuticle is arranged in a laminar fashion (indicated by bracket in Fig. 8), while in the protruding bulge regions procuticular chitin does not adopt a preferred organization. In *TcKnk2* dsRNA-treated insects, an amorphous electron-dense material occasionally accumulates within the protruding denticle region (Fig. 8; panel D4, black arrows). This phenotype is reminiscent of that observed in insects injected with dsRNA for *TcKnk* (Fig. 8; panel D2). However, simultaneous knockdown of *TcKnk2* with *TcCht5* transcripts rescued the Velcro-denticle phenotype with the disappearance of the electron dense materials (Fig. 8; panel D5). *TcKnk3-3′* dsRNA-treated insects did not exhibit any obvious phenotype in the denticles (Fig. 8; panel D6 and D7).

dsRNA treatment for *TcKnk2* perturbed the organization of the taenidial cuticle and showed accumulation of electron dense material within the taenidial procuticle (Fig. 8, panel T4 black arrow head). Similar results were also seen after RNAi for *TcKnk3-3′* (Fig. 8, panel T6, black arrow head). Upon simultaneous down regulation of *TcKnk2* and *TcCht5, or TcKnk3-3′* and *TcCht5*, very little electron-dense material was observed in the respective taenidial procuticle and the tracheal shape was also recovered, indicating rescue of the phenotype (Fig. 8, panel T5 and T7). Taken together, our ultrastructural analysis indicates that like TcKnk, both TcKnk2 and TcKnk3 are involved in protection of chitin in the “Velcro denticles” and tracheae from chitinase. It is interesting to observe that *TcKnk2* and *TcKnk3-3′* dsRNA treatment defects are associated with structures in which chitin is normally not organized in a laminar fashion. The lethal phenotypes observed at the pharate adult stage of molting after *TcKnk2* and *TcKnk3-3′* RNAi could result from loss of integrity of body wall denticles and a failure to develop tracheal taenidia normally. These results indicate significant roles for TcKnk-like proteins in chitin maintenance and cuticle integrity in the body wall denticles and *tracheae*.

## Discussion

### Insect *Knk* Gene Family Has Three Members

Even though the *knickkopf* mutation was described as early as 1984 [12], its molecular characterization was not accomplished until much later, when Ostrowski et al., [13] identified the gene associated with the *knk* mutation characterized by the “broken-head” and the “blimp” phenotype at the embryonic larval stage. This gene was also shown to be essential for organization of the filamentous chitin structures during tracheal tubule growth and cuticle differentiation [5]. Our recent study has demonstrated an important role for TcKnk in protecting the newly synthesized procuticular chitin from degradation by active chitinases by co-localizing with chitin in the procuticle [11]. Additionally, TcKnk was shown to bind to chitin and to be important for the laminar organization of procuticular chitin in elytral and body wall cuticles of *T. castaneum*.

During a bioinformatics search of the genomes of insect species that have been fully sequenced and annotated, we came across two other paralogs of the *TcKnk* gene, which have not been studied so far in any insect species. Domain analysis of TcKnk-like proteins revealed a similar domain organization compared to TcKnk. Like TcKnk, TcKnk2 and TcKnk3 also have two N-terminal DM13 domains, one DOMON domain in the middle and a C-terminal domain that is highly conserved in all three members of this family of Knk proteins. Although the functions of the DM13 and DOMON domains are unclear, they have been predicted to have important roles in redox or electron transfer reactions [17]. The DOMON domain is also predicted to bind with heme or sugars [18]. In a recent work it was demonstrated that residues in the Drosophila Knk DOMON domain predicted to be important for substrate binding are essential for viability [19]. Biochemical studies showed that TcKnk extracted or released from cells has a very strong affinity for colloidal chitin [11].

### Knk Paralogs Are Found In *Insecta* But Not In Arthropods

The widespread occurrence and retention of orthologs of *TcKnk2* and *TcKnk3* genes in other insect orders belonging to hemipteran, dipteran, lepidopteran and hymenopteran lineages indicates that duplication of the ancestral *Knk* gene from which these paralogs were derived must have occurred before the branching of these orders and that these *Knk*-like genes probably perform essential functions. While they are present in several insect orders, their absence in the deer tick, water flea and nematodes, which have only one *Knk* homolog, suggests that the ancient *Knk* gene has undergone gene duplication in the progenitor of those insect orders and that the insect paralogs assumed specialized functions in different cuticle-forming tissues.

### Knk Paralogs Have Distinctive And Essential Functions In Insect Cuticle Development

RNAi studies for *TcKnk2* and *TcKnk3* confirmed the importance of these genes for cuticle morphogenesis apparently not fulfilled by *Knk* alone. *TcKnk2* dsRNA treatment at larval stages led to molting arrest at the pharate adult stage of development in a majority of the animals. *TcKnk3-3′* transcript (but not the full length or 5′-transcripts) also appears to be essential for molting and survival during the pupal adult transformation. The finding that molting defects were observed after administration of dsRNAs for *TcKnk2* and *TcKnk3-3′*, even though these dsRNAs did not result in depletion of the *TcKnk* transcripts, reveals specialized functions for *TcKnk2* and *TcKnk3* during cuticle morphogenesis, which are not redundant with those of *TcKnk*.

Unlike *TcKnk*, which is required for every molt, *TcKnk2* and *TcKnk3* have essential roles only during morphogenesis to the adult stage. Earlier molts do not seem to be affected. *TcKnk* has been shown to be important for arranging the chitin into laminae in the elytral and adult abdominal body wall cuticles in *T. castaneum* and in the larval cuticle in *D. melanogaster* [11],[14]. We report here that *TcKnk* is also required for maintenance of the normal shape of the denticles and the tracheal taenidiae. While TcKnk affects the total chitin content dramatically both at the pharate pupal and pharate adult stages [11], RNAi of the two paralogous *TcKnk*-like genes are not manifested in altered chitin content at the pharate pupal stage, consistent with the absence of observable effects on molting or morphology at the larval-to-pupal molt. Even though there was a statistically significant reduction in total chitin content after RNAi for *TcKnk2* as well as *TcKnk3-3′* at the pharate adult stage, we could not detect changes in the laminar architecture of the elytral procuticle or body wall cuticle as has been demonstrated following *TcKnk* RNAi [11]. Therefore, we conclude that these two paralogous *Knk* genes are not required for organization of the elytral cuticle or the body wall cuticle. On the other hand, TEM analysis of denticles in the lateral body wall and tracheae of *TcKnk2* and *TcKnk3-3′* dsRNA-treated pharate adult insects showed accumulation of electron-dense material (probably proteins) in the chitin matrix of Velcro-denticle bulges and the taenidia, suggesting disorganized procuticles in these structures. Therefore, *TcKnk*-like genes may have a secondary role in cuticular chitin maintenance and organization in specialized cuticle-forming tissues such as those involved in the formation of the denticles and tracheal taenidia. The accumulation of electron-dense material in tracheae after *TcKnk2* and *TcKnk3* dsRNA treatments indicates a possible role for TcKnk-like proteins in influencing proper distribution of putative cuticular proteins into the tracheal cuticle.

In a minority of insects after RNAi for *TcKnk2* and *TcKnk3*, wrinkled elytra, dimpled pronotum and wing defects were observed at the macroscopic level in spite of the presence of normal laminar architecture at the TEM level (Fig. 7). There are many potential causes of gross elytral malformations beyond disruption of laminar architecture as we have reported previously in insects after RNAi of genes for neuropeptides and their receptors [20]. Even something so simple as an adhering fragment of pupal exuvium that impedes complete adult emergence and subsequent expansion of elytra can result in elytral malformations [21].

Only Knk and Knk3-FL-2 are predicted to have a PI-PLC-cleavable GPI anchor and, therefore, are expected to be released to the procuticle. We have previously demonstrated that the Knk protein is indeed cleaved by PI-PLC and is associated with chitin throughout the elytral and body wall procuticles and that this association is essential for protection of chitin from molting fluid chitinases [11]. Because *TcKnk3*-3′ transcripts containing exon 8a sequences are also predicted to encode a protein containing the cleavable GPI-anchor, Knk3-protein may also associate with chitin in specialized cuticles such as body wall “Velcro denticles” and the taenidia. The reduction in chitin and the restoration of the chitin content following knock-down of the major chitinase, Cht5, is consistent with such a role for the *Knk3-3′* transcript-derived protein. On the other hand, Knk2 has a C-terminal trans-membrane domain and topological predictions indicate that the rest of the protein will be exposed to the extracellular side. The restoration of chitin levels in insects following down-regulation of both *TcKnk2* and *TcCht5* transcripts suggests that Knk2 protein may also protect chitin. Whether this protection requires release of this protein from the membrane is unresolved.

### Only The *Tcknk3*-3′ Transcripts With Exon 8a Are Indispensable For Insect Survival

The detection of multiple alternatively spliced and polyadenylated variants for the transcripts of *TcKnk3*, named *TcKnk3-FL* (with and without exon 8a), *TcKnk3-5′* and *TcKnk3-3′* (with and without exon 8a) complicated our functional analysis of the transcripts for this gene. Our most important finding was that RNAi of *TcKnk3* transcripts using exon 8a dsRNA resulted in molting arrest and lethality at the pharate adult stage. RT-PCR analysis indicated that this dsRNA treatment did not affect the levels of transcripts without exon8a. Thus it appeared that either full-length transcripts with exon 8a and/or truncated transcripts with exon 8a may be the only transcripts essential for survival and molting at the pharate adult stage. On the other hand, a nearly complete loss of the *TcKnk3-FL* transcript (along with the *TcKnk3*-5′-transcripts), brought about using dsRNA for exon 5 (or upstream exons) of this gene, did not result in mortality or any visible phenotypes indicating that none of the longer transcripts (and the *TcKnk3*-5′-transcripts) and their translation products are essential for survival and/or molting. Therefore, the lethality associated with the loss of transcripts with the exon 8a sequences must be entirely due to short transcripts (i.e. lacking exons 1 through 5). Unfortunately we were unable to determine the precise start point of this short transcript because the only region where we could design the reverse primer for the 5′-RACE is the 55 nucleotide-long exon 8a. 5′-RACE reactions using two reverse primers from exon 8a- and from exon 9 failed to identify the transcription start point even when we used total RNA depleted of *TcKnK3* full length and *5′*-transcripts (using dsRNA for exon 5) as the template. We believe that this transcript starts downstream of exon 5 based on the RNAi results described earlier. Other strategies for identifying the start site of TcKnk3-3′ transcripts using forward primers designed in intron 5 also were unsuccessful.

The inclusion of exon 8a results in a shift of the open reading frame. As shown in Fig. 1, the protein is altered to have a unique C-terminus that includes a GPI anchor. Transcripts without exon 8a predominate at most developmental stages (see Fig. S1). The exception is during the mid-pupal stage when the isoform of the Knk3 protein encoded using exon 8a is expected to peak. This is on or around pupal day 2–3, which just precedes the time point when developmental arrest occurs using exon 8a dsRNA. Interestingly, *TcKnk3* orthologs of other insect species are predicted to encode proteins that are highly similar to this long additional stretch of >400 amino acids found in TcKnk3 protein derived from read-through of exon 8a. We have carried out a careful analysis of preview data from the developmental stage time course transcriptional profiling with RNA-seq in *D. melanogaster* (modENCODE Project led by Sue Celniker [22]) publicly available in Flybase (http://flybase.org/cgi-bin/gbrowse/dmelrnaseq/). RNA Seq data of the DmKnk3 ortholog, Skeletor R-E, supports the notion that a similarly placed 55 nucleotide-long exon equivalent to TcKnk3 exon 8 in this gene leads to variation in the relative abundance of transcripts with and without this putative alternatively spliced exon in this dipteran species as well.

It should be pointed out that the truncated TcKnk3 protein derived from the shorter transcript with exon 8a sequences will be missing the two N-terminal DM13 domains but still have the DOMON domain, exon 6 through 8 derived sequences and additional exon 9-encoded sequences at the C-terminus derived from read-through of exon 8a. This read-through will not occur in transcripts that are devoid of exon 8a because the ribosomes encounter an in-frame stop codon very close to the beginning of exon 9. Since this longer protein is predicted to have a GPI anchor, it may be destined for transport to the plasma membrane. At present, we have been unable to identify whether it has an N-terminal signal peptide sequence to allow this protein to enter the ER. Absence of an antibody specific for this protein also precludes determination of the precise cellular location of this protein at present. Since the TcKnk3-FL-1 protein is the predominant form at most developmental stages and yet appears to be dispensable for survival, the sequences present in the C-terminal part of the TcKnk3 protein and/or the presence of the C-terminal GPI anchor may be critically important for metamorphosis at the pharate adult stage.

### Alternative Splicing Of Exon 8a Of Tcknk3 Is Developmentally Regulated

Of the three members of the *Knk* gene family, only *Knk3* is known to have the potential to give rise to alternatively spliced transcripts. There is no experimental evidence for additional transcripts for *TcKnk* and *TcKnk2* genes based on 5′-RACE, 3′-RACE or RT- PCR. The finding that *TcKnk3-5′* and *TcKnk3-3′* transcripts appear only when the full-length transcript is down-regulated (Fig. 2) raises the interesting possibility that there may be physiologically important regulatory mechanisms that control the appearance and relative amounts of these alternatively spliced transcripts. The much higher levels of accumulation of the *TcKnk3-3′* transcript compared to the *dsRNA TcVer*-treated control, when dsRNA for exon 5 is administered to the larvae, is consistent with a regulatory mechanism that controls the position of the transcription start either upstream of exon 1 or downstream of exon 5. Indeed a similar situation has been reported in the case of *D. melanogaster* ortholog of *TcKnk3* gene (named *Skeletor*), which is known to yield multiple transcripts [23]. In this study, we have demonstrated that additional complexity arises by the inclusion or exclusion of exon 8a sequences, which changes the reading frame allowing the production of a longer protein with additional C-terminal sequences. The changes in the relative amounts of transcripts with and without exon 8a during various stages of development and especially during the pupal stages indicates additional control at the level of splicing of pre-mRNA presumably through an RNA-binding protein. A similar alternative splicing mechanism that alters the ratios of alternatively spliced transcripts during the pupal stage occurs in the case of insect chitin synthase-A genes (*Tribolium castaneum CHS-A* exon 8b and *Manduca sexta CHS-A* exon 18b) in which transcripts with alternative exon b are known to accumulate during pupal stages and especially in the tracheae [21],[24],[25].

### Tcknk2 And Tcknk3 Have A Role In Shaping Non-Laminar Cuticle

It is likely that specific splice forms of *TcKnk3* accumulate in tissues making specialized cuticles such as velcro denticles and taenidia of tracheae. The finding that RNAi of either *TcKnk2* or *TcKnk3-3′* does not affect the laminar organization of chitin in the elytral and body wall cuticle is consistent with this idea of specialization among different paralogous members of the Knk family. In the body wall cuticle that underlies the denticles, the laminar organization of chitin also appears unaffected after RNAi for *TcKnk2* and *TcKnk3-3′*. However, the bulged region of velcro denticles exhibit accumulation of electron dense material (presumably cuticular proteins) in these specialized cuticle-forming structures. The restoration of normal ultrastructure of the denticles and the taenidia following RNAi for both *TcKnk2* (or *TcKnk*3) along with *TcCht5* indicates that these two orthologous proteins also act in a manner similar to TcKnk in binding and protecting chitin. In these specialized structures, in addition to the parallel layers of chitin laminae, there are additional layers of chitin fibers whose orientation follows the shapes of denticles or taenidia. Knk2 (predicted to be membrane-bound) and the Knk3-3′ protein, which is predicted to have a cleavable GPI anchor, may be essential for forming these specialized cuticular structures. The level of expression in these tissues, their precise locations, and how Knk and the Knk-like proteins interact with each other will be interesting points of future studies.

## Materials And Methods

### Insect Cultures

*T. castaneum* GA-1 strain was used for all experiments. Insects were reared at 30°C in wheat flour containing 5% brewer's yeast under standard conditions as described previously [26].

### Identification Of *Tcknk*-Like Genes In The *T. Castaneum* Genome Database

A genome-wide TBLASTN search using the amino acid sequence of *T. castaneum* Knk (TcKnk) as the query was carried out at NCBI (http://www.ncbi.nlm.nih.gov/) and BeetleBase (http://beetlebase.org/). This resulted in identification of two genes, which we have named *T. castaneum Knk-2* (*TcKnk2*) and *T. castaneum Knk-3* (*TcKnk3*).

### Identification Of *Tcknk*-Like Genes In Insect Genomes

Using the amino acid sequences of TcKnk2 and TcKnk3 as queries, orthologs for *TcKnk*-like genes were detected in all of the fully sequenced insect genomes. A second round of “BLAST” searches with the amino acid sequences of these Knk-like proteins from insects failed to identify additional *Knk*-like genes in insect genomes.

### Cloning And Sequencing Of Cdnas For *Tcknk*-Like Genes

The complete coding sequences of *TcKnk2* and *TcKnk3* were amplified using gene-specific primers (Table S1) and cDNA prepared from RNA extracted from whole insects at the pharate adult stage of beetle development. 5′- and 3′-RACE reactions were performed to determine the upstream and downstream untranslated regions for both of these genes. The sequences of the full-length transcripts for *TcKnk2* and *TcKnk3* were deduced by combining the data from the above-mentioned experiments. Using a pair of forward and reverse primers derived from the 5′ and 3′-ends of the predicted mRNA, full-length cDNAs were amplified by PCR and cloned into pGEMT vector (Promega). Cloning of cDNAs for *TcKnk3* using the same techniques resulted in isolation of several alternatively spliced variants, which are described in the Results section. Sequencing of all cDNA clones was carried out at the DNA sequencing facility at Kansas State University.

### Phylogenetic Analysis Of Tcknk-Family Proteins

Multiple sequence alignment of TcKnk-family proteins from insects was carried out using the ClustalW software prior to phylogenetic analysis. MEGA 4.0 [16] was used to construct the consensus phylogenetic tree, using the neighbor-joining method. To evaluate the branch strength of the phylogenetic tree, bootstrap analysis of 5,000 replications was performed.

### Developmental And Tissue-Specific Expression Profiles For *Tcknk*-Like Genes

To determine the developmental stage-specific expression profiles, total RNA was extracted from embryos, young larvae, last instar larvae, pharate pupae, pupae, young adults (0 d-old) and mature adults (10 d-old) using the RNeasy Mini kit (Qiagen). For determination of tissue specificity of expression, total RNA was also isolated from midgut, hindgut and carcass (whole body minus gut) of last instar feeding stage larvae (n = 10) according to the manufacturer's instructions. The Superscript III first–strand synthesis system for RT-PCR (Invitrogen) was used to synthesize first-strand cDNA according to the manufacturer's instructions. Gene-specific primers were used to detect each transcript from the prepared sets of cDNAs (Table S1). A *TcRpS6* (*T. castaneum ribosomal protein S6*) cDNA fragment was amplified using a pair of primers and served as an internal loading control for RT-PCR [27].

### Rna Interference Studies

Two regions from two different parts of *TcKnk2* gene with the greatest sequence divergence were selected as targets for RNAi (Table S1). A total of nine dsRNAs were designed for achieving RNAi of one (or more) of the three different transcripts of *TcKnk3* by targeting different exons/introns (exons 1, 2, 3, 5, 6, 7-5′-terminal, 7-3′-terminal, 8, 9 and exon 8a). Pairs of forward and reverse primers corresponding to these regions with additional T7 promoter sequences at the 5′-ends were synthesized (Table S1) and used for the preparation of dsRNAs using an Ampliscribe T7-Flash Transcription Kit (Epicentre Technologies) as described previously [21]. A dsRNA for the gene responsible for eye pigmentation named *T. castaneum Vermilion* (*TcVer*) was used as a control for monitoring non-specific effects of dsRNA administration and for assessing the efficiency of RNAi. The purified dsRNAs were injected into penultimate instar larvae, last instar larvae and pharate pupae (200 ng per insect, n = 40). After 4–5 d, total RNA was extracted from pools of five insects at either pupal d 3 (for *TcKnk2*) or the pharate adult stage (for *TcKnk3*) for measuring transcript levels by RT-PCR using gene-specific primer-pairs. The remaining insects were observed daily for any visible abnormalities and mortality.

### Northern Blot Analysis For Detection Of *Tcknk3* Transcripts

Total RNA was extracted from pharate adult insects (n = 4) treated with *TcVer*, *TcKnk3-exon9-* and *TcKnk3-exon5*-specific dsRNAs at the prepupal stage. Ten µg RNA samples were subjected to gel electrophoresis and transferred onto a nitrocellulose membrane. ^32^P-labeled *TcKnk3*-5′- and 3′-terminal DNA probes were designed using the primers listed below. ^32^P-labeled *TcKnk3*-5′-terminal probe was prepared from a 551 bp DNA fragment amplified by using exon 1-specific forward primer (5′-ATGGGCCCCATCGTTGCATT-3′) and exon 5-specific reverse primer (5′-GCGAAATTCTGGGTGGGTCG- 3′). *TcKnk3*-3′-terminal probe was prepared from a 1,370 bp DNA fragment obtained by using an exon 8-specific forward primer (5′-CACGACAAGTGCGACGAGCA-3′) and exon 9-specific reverse primer (5′-GCCGCGGAACTTATCAAAGC- 3′). Duplicate blots were hybridized with ^32^P-labeled *TcKnk3*-5′- or 3′-terminal probe at 65°C overnight. High stringency hybridization and washing conditions were employed. Transcripts of differing sizes were then detected after autoradiography using an imaging plate and Typhoon scanner.

### Chitin Content Analysis

*TcKnk2-* or *TcKnk3* (exon-9)-specific dsRNAs were injected into last instar larvae and pharate pupae (n = 20). Four to five days after injections, insects were collected at pharate pupal and pharate adult stages of development. Three days after administration of dsRNA for *TcKnk3* to 20 female adult beetles, mating was carried out with an equal number of untreated adult males and batches of ∼200 embryos were collected every three days. Total chitin content analysis of whole insects (n = 5) collected at the indicated stages was performed as described previously [28]. GraphPad Prism software was used to plot the graphs and for data analysis.

### Transmission Electron Microscopic (tem) Analysis

For TEM analysis, insects were injected with *TcKnk2-* or *TcKnk3* (exon-9)-specific dsRNAs at pharate pupal stages of development. Five days later, pharate adult insects were collected and fixed overnight at room temperature using a fixative containing 2% para-formaldehyde and 2% glutaraldehyde in 0.1 M sodium cacodylate buffer (pH 7.4). Samples were sectioned to obtain 70 nm thin sections, processed and observed under TEM for final imaging as described previously [11].

## Supporting Information

Figure S1 Developmental stage-specific and pupal day-specific expression of *TcKnk3*-*FL-1* and *TcKnk3*-*FL-2* by RT-PCR. (A) Developmental expression profiles of *TcKnk3*-*FL-1* and *TcKnk3*-*FL-2* transcripts. cDNAs were prepared from total RNA extracted from whole insects at several developmental stages including E, embryos; YL, young larvae (penultimate instar or younger); ML, mature larvae; PP, pharate pupae; P, pupae; YA, young adults (0 d- old); A, mature adults (10 d-old). (B) Pupa day-specific expression of *TcKnk*-like genes in the feeding stage last instar larvae. P0 to P5, Pupa day 0 to pupa day 5; A0, Adult day 0. RT-PCR for the *TcRpS6* transcripts was carried our prior to these analyses to ensure that equal amounts of cDNA templates from different developmental stages were being used in these comparisons. Results are from 28, 28 and 24 cycles of RT-PCR for *TcKnk3-FL-1 and TcKnk3*-*FL-2 genes* and *TcRpS6*, respectively. The grainy quality of the amplification products relative to the RpS6 RT-PCR products is due to different camera settings. (TIF)

Figure S2 Amino acid sequence alignment for TcKnk3-FL-1 and TcKnk3-FL-2. Black shading of amino acid residues indicates identity. (TIF)

Figure S3 Sequence alignment of TcKnk-family proteins. Black and gray shading of amino acid residues indicates identity. Underlines and boxes of different colors indicate different domains: green, signal peptide; blue, DM13 domains; red, dopamine monooxygenase N-terminal like (DOMON) domain; orange underline, GPI anchor-specifying sequence; black underline, transmembrane domain. 149 amino acids were removed from the C-terminus of TcKnk3-5′ protein for alignment. (TIF)

Figure S4 Specificity of dsRNA-mediated down-regulation of *TcKnk3* transcripts. Levels of *TcKnk3-FL* and *TcKnk3-5′* transcripts after treatments with different dsRNA were determined by RT-PCR (28 cycles) using appropriate primers designed to amplify the full length or the 5′-TcKnk3 cDNA products. Indicated dsRNAs (labeled at the top of each panel) were injected into pharate pupal insects. Five days post-injection, RNA was collected from pharate adult insects for cDNA preparations. *TcRpS6* (24 cycles) was used as an internal loading control for RT-PCR. TcVer dsRNA treatment was used as a control. (TIF)

Table S1 Primer sequences. (DOCX)

Table S2 Summary of phenotypes observed after *TcKnk3* dsRNA-treatments. (DOC)
