# Supplementary figures and images for: Functional Specialization Among Members Of Knickkopf Family Of Proteins In Insect Cuticle Organization
Source: PLoS Genet. 2014 Aug 21;10(8):e1004537. doi: 10.1371/journal.pgen.1004537 (PMC4140639; doi:10.1371/journal.pgen.1004537)

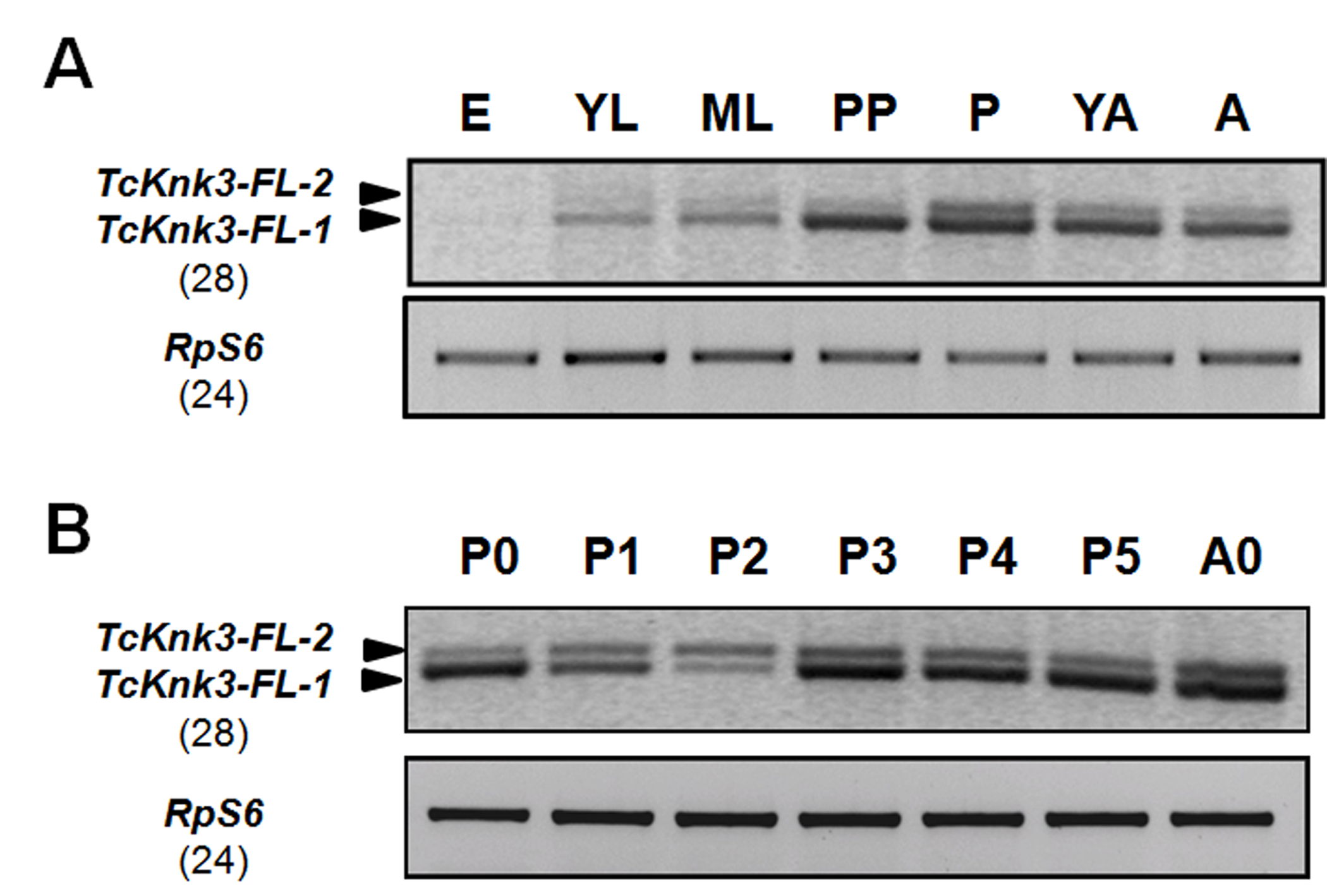

Supplement: Figure S1 — (TIF) [file pgen.1004537.s001.tif]

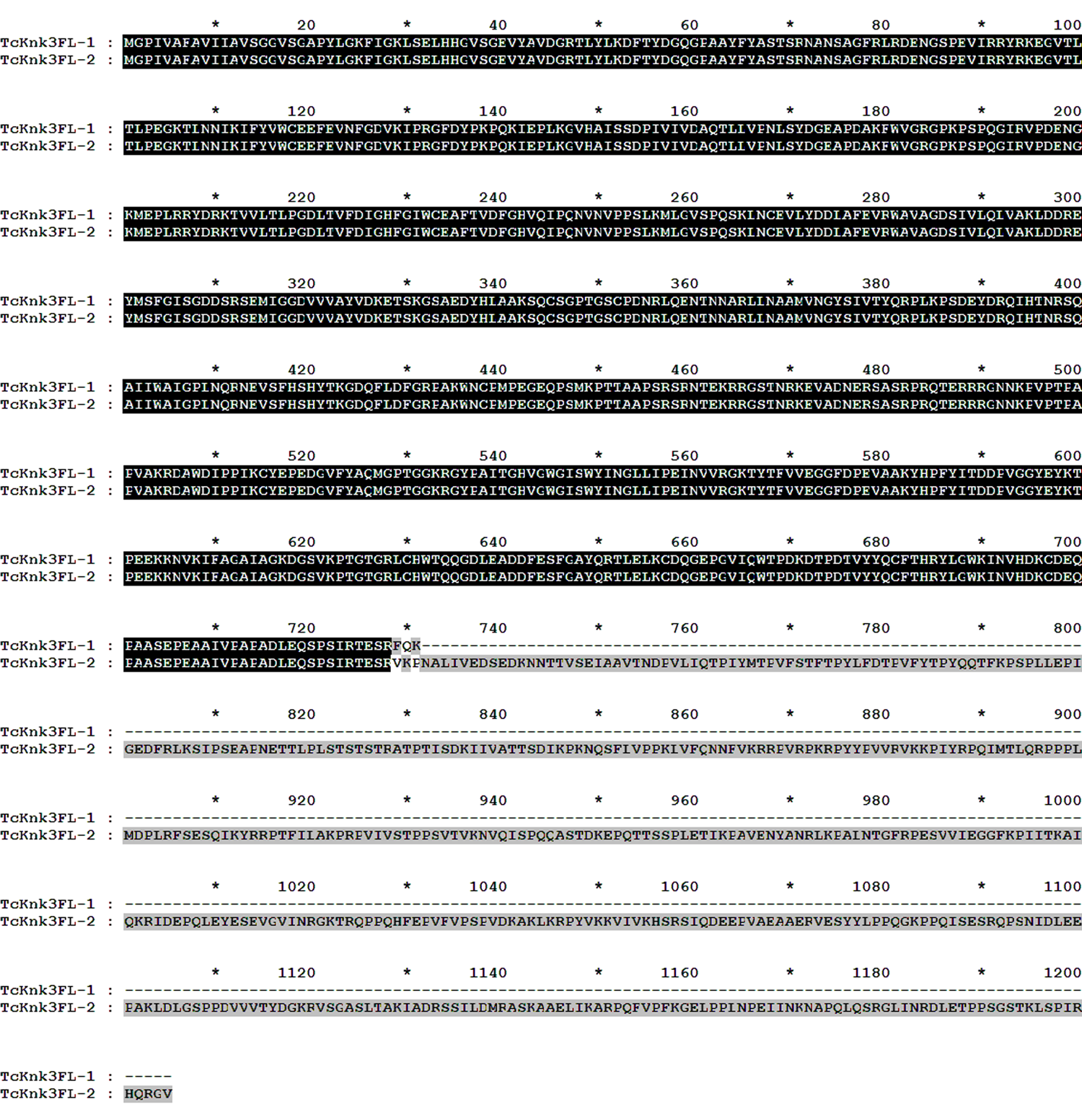

Supplement: Figure S2 — (TIF) [file pgen.1004537.s002.tif]

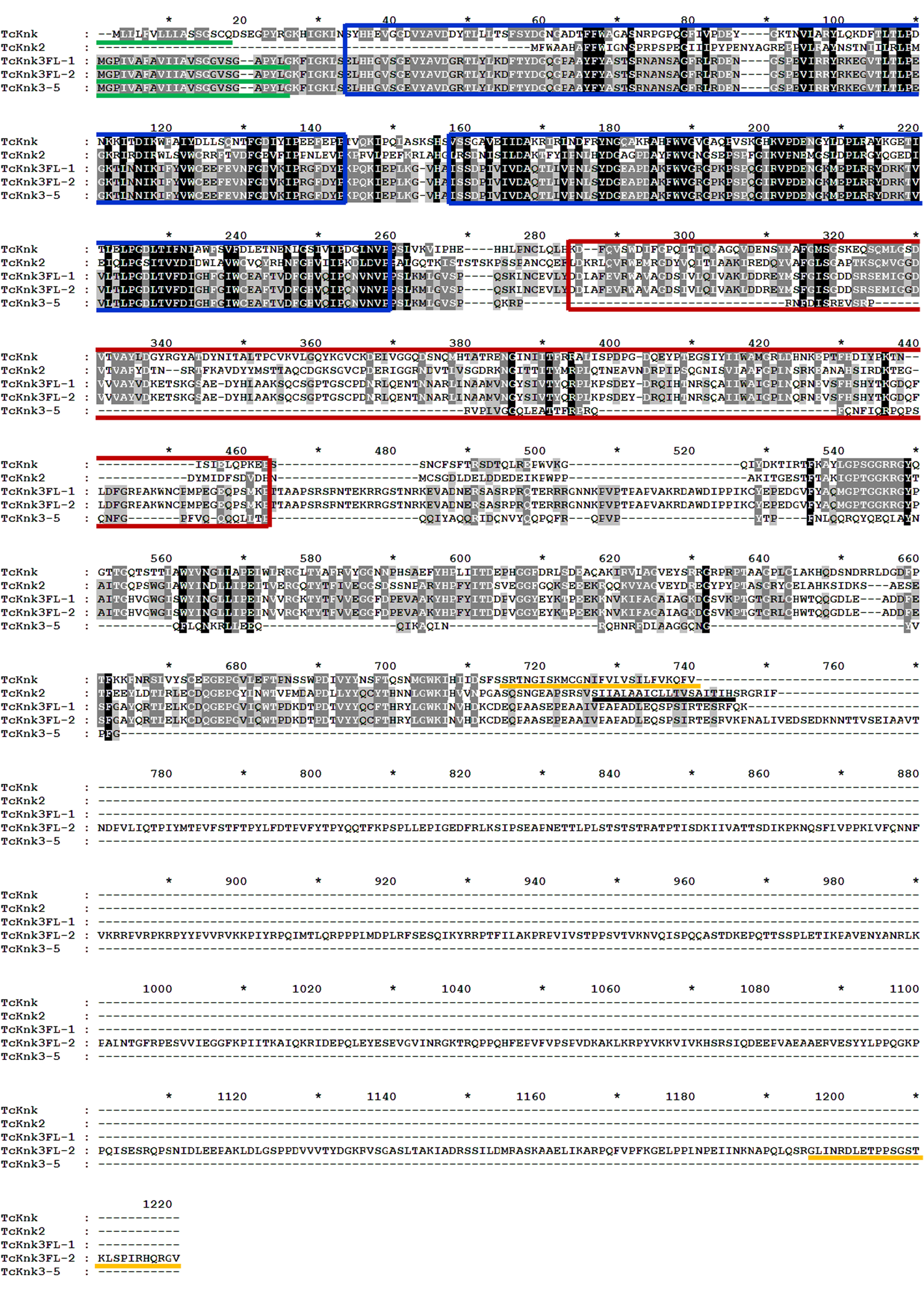

Supplement: Figure S3 — (TIF) [file pgen.1004537.s003.tif]

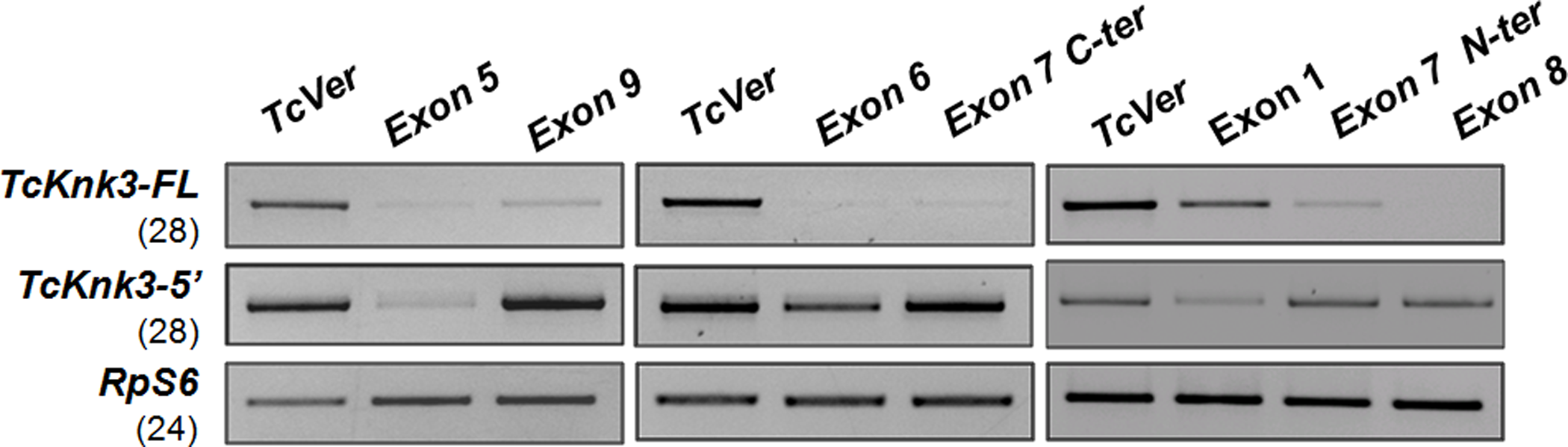

Supplement: Figure S4 — (TIF) [file pgen.1004537.s004.tif]
