# Supplementary material for: Functional Specialization Among Members Of Knickkopf Family Of Proteins In Insect Cuticle Organization
Source: PLoS Genet. 2014 Aug 21;10(8):e1004537. doi: 10.1371/journal.pgen.1004537 (PMC4140639; doi:10.1371/journal.pgen.1004537)
Supplement: Table S1 — (DOCX) [file pgen.1004537.s005.docx]

Table S1. Primer sequences

| **Primer** | **Forward Primer (5’-3’)** | **Reverse Primer (5’-3’)** |
| --- | --- | --- |
| TcKnk2 | **CGTGAAGATCAATACGTCGC** | **GGAGCGTCTCGATAATTGCA** |
| TcKnk2 dsRNA1 | **TAATACGACTCACTATAGGGTGGCAGCGAAAATCCG** | **TAATACGACTCACTATAGGGTTGTCCCGACGAACGC** |
| TcKnk2 dsRNA 2 | **TAATACGACTCACTATAGGGTCATCACCACCATCAC** | **TAATACGACTCACTATAGGGTGAGTTGGACGACGAG** |
| TcKnk2 R1-5’Race- 1 | **----** | **CTTCAAAGCCGTCAAGTCCT** |
| TcKnk2 R2-5’Race-2 | **----** | **CGAGGACATCGAGATCCAGC** |
| TcKnk2 R3-5’Race-3 | **----** | **CCTAAGACTGCCGATGGGTA** |
| TcKnk2-F1- 3’-In | **----** | **CGCTCATTCCATAAGAGACA** |
| TcKnk2-F1- 3’-Out | **----** | **GCACCTTCACGGCGAAAATC** |
| TcKnk3-FL | **ATGGGCCCCATCGTTGCATT** | **GTTTTGTGTCGTTATTTTGTG** |
| TcKnk3-5’ | **ATGGGCCCCATCGTTGCATT** | **GGCGAAGTGGTTCAGGTGTT** |
| TcKnk3 R1-5’ Race -1 | **----** | **CGCCGGAGACAGTATTGTTT** |
| TcKnk3 R2-5’ Race -2 | **----** | **GCGAAATTCTGGGTGGGTCG** |
| TcKnk3 R3-5’ Race-3 | **----** | **CCAGAAGATCGAGCCTTTGA** |
| TcKnk3-F1- 3’ Race - 1 | **GAAGAAAAATGTGAAGATTTTC** | **----** |
| TcKnk3-FL- 3’ Race - 2 | **GACGGAAGTGTTAAACCTACAG** | **----** |
| TcKnk3-FL- 3’ Race- 3 | **CAAGGCGATTTGGAAGCCGATG** | **----** |
| TcKnk3- exon 8a R | **----** | **TTATCTTCTGAATCTTCAAC** |
| TcKnk3- exon 8a R1 | **----** | **ATTAAAGCATTTGGTTTTAC** |
| TcKnk3- dsExon 1 | **TAATACGACTCACTATAGGGTGAAAGACATCGAAGG** | **TAATACGACTCACTATAGGGTTATCGCCGTTTCAGG** |
| TcKnk3- dsExon 2 - 3 | **TAATACGACTCACTATAGGGTCCGGCGCTCCCTATT** | **TAATACGACTCACTATAGGGTCGTGTGGTGCGAGGA** |
| TcKnk3- dsExon 5 | **TAATACGACTCACTATAGGGTCCTGACAGTGTTCGA** | **TAATACGACTCACTATAGGGTCACAGTCGAAGCTGA** |
| TcKnk3- dsExon 6 | **TAATACGACTCACTATAGGGTTCGAAGCTGAACTGC** | **TAATACGACTCACTATAGGGTCAACTAGTTGCAAAATTAG** |
| TcKnk3- dsExon 7N-ter | **TAATACGACTCACTATAGGGTGGCATCTCGGGCGAT** | **TAATACGACTCACTATAGGGTGACCGGCAGATCCAC** |
| TcKnk3- dsExon 7C-ter | **TAATACGACTCACTATAGGGTCCCTTCTACATCACC** | **TAATACGACTCACTATAGGGTCGGATAAAGACACTCC** |
| TcKnk3- dsExon 8 | **TAATACGACTCACTATAGGGTGTTTCACGCATCGTT** | **TAATACGACTCACTATAGGGTCGGACCGAATCAAGG** |
| TcKnk3- dsExon 8a | **TAATACGACTCACTATAGGGTCAAGGGTAAAACCAA** | **TAATACGACTCACTATAGGGTTACAACAGTTTCAGAAA** |
| TcKnk3- dsExon 9 | **TAATACGACTCACTATAGGGTCCACAGCACTTCGAA** | **TAATACGACTCACTATAGGGTGTAAGCGAGTTTCCG** |
