# Supplementary material for: Functional Specialization Among Members Of Knickkopf Family Of Proteins In Insect Cuticle Organization
Source: PLoS Genet. 2014 Aug 21;10(8):e1004537. doi: 10.1371/journal.pgen.1004537 (PMC4140639; doi:10.1371/journal.pgen.1004537)
Supplement: Table S2 — (DOC) [file pgen.1004537.s006.doc]

**Table S2. Summary of phenotypes observed after *TcKnk3* dsRNA-treatments.**

| Exon Number | Terminal phenotype stage | % Mortality | % Adult “weak” phenotype |
| --- | --- | --- | --- |
| Exon 1 | None | --- | --- |
| Exon 2 and 3 | None | --- | --- |
| Exon 5 | None | --- | --- |
| Exon 6 | Pharate adult | 100 | --- |
| Exon 7 5’-terminal | None | --- | --- |
| Exon 7 3’-terminal | None | --- | --- |
| Exon 8 | Pharate adult | 82 | 18 |
| Exon 8a | Pharate adult | 66 | 15 |
| Exon 9 | Pharate adult | 100 | --- |
